# Pre-treatment monocytic myeloid-derived suppressor cells as predictive biomarkers for immune checkpoint inhibitor response in clear cell renal cell carcinoma

**DOI:** 10.3389/fimmu.2025.1641383

**Published:** 2025-08-21

**Authors:** Yiming Jin, Hisamitsu Ide, Masayoshi Nagata, Takuro Kobayashi, Jun Lu, Yoshihiro Ikehata, Fumitaka Shimizu, Yan Lu, Shigeo Horie

**Affiliations:** ^1^ Department of Urology, Graduate School of Medicine, Juntendo University, Tokyo, Japan; ^2^ Department of Molecular and Medical Pharmacology, David Geffen School of Medicine, University of California, Los Angeles, Los Angeles, CA, United States; ^3^ Department of Innovative Longevity, Graduate School of Medicine, Juntendo University, Tokyo, Japan; ^4^ Data Science and Informatics for Genetic Disorders, Graduate School of Medicine, Juntendo University, Tokyo, Japan

**Keywords:** clear cell renal cell carcinoma, myeloid-derived suppressor cells, immune checkpoint inhibitors, biomarkers, elastic net regression

## Abstract

**Background:**

Immune checkpoint inhibitors (ICIs) are a cornerstone of systemic therapy for clear cell renal cell carcinoma (ccRCC), yet response rates remain variable and predictive biomarkers are lacking. This study aimed to determine whether baseline levels of myeloid-derived suppressor cells (MDSCs), especially monocytic (M-MDSC) and polymorphonuclear (PMN-MDSC) subtypes, could predict ICI response in ccRCC patients.

**Methods:**

In this prospective cohort study, 20 ccRCC patients receiving ICI-based therapy for at least 3 months were enrolled. Peripheral blood mononuclear cells were collected before and after treatment to quantify total MDSCs, M-MDSCs, and PMN-MDSCs via flow cytometry. Additional clinical variables, including blood cell counts and metabolic profiles, were assessed. Elastic net regression identified key variables associated with treatment response. Multivariable logistic regression was used to evaluate their predictive value. The primary outcome was objective response (CR/PR) based on RECIST 1.1.

**Results:**

Among 47 clinical and laboratory variables with an area under the curve (AUC) greater than 0.6, elastic net regression identified 7 key predictors of immunotherapy response. Notably, higher baseline levels of monocytic MDSCs (M-MDSCs) and their proportion within total MDSCs were independently associated with objective response to immune checkpoint inhibitors (OR = 3.082, p = 0.041 and OR = 5.764, p = 0.036, respectively). Following treatment, responders exhibited a significant decline in circulating M-MDSC levels, whereas non-responders did not.

**Conclusions:**

Baseline circulating M-MDSC levels and their relative proportion within total MDSCs may serve as potential predictive biomarkers for response to immune checkpoint inhibitors in ccRCC patients. These findings highlight the role of MDSCs in modulating immunotherapy efficacy and suggest their clinical utility in guiding personalized treatment strategies.

## 1 Introduction

Clear cell renal cell carcinoma (ccRCC) accounts for approximately 70%–80% of renal cell carcinoma (RCC) cases and is characterized by mutations in the Von Hippel-Lindau (VHL) gene, leading to dysregulation of the hypoxia-inducible factor (HIF) pathway, enhanced angiogenesis, and an immunosuppressive tumor microenvironment (TME) (1, 2). In recent years, immune checkpoint inhibitors (ICIs), particularly those targeting PD-1/PD-L1 pathways, have significantly improved the treatment of metastatic ccRCC (mccRCC), replacing VEGF-TKIs as the preferred first-line therapy either as monotherapy or in combination with VEGF-TKIs or CTLA-4 inhibitors (3).

Despite significant clinical benefits, ICI responses vary markedly among mccRCC patients, with objective response rates ranging from 40% to 60% (4, 5). Robust predictive biomarkers remain lacking, complicating patient selection and treatment optimization (6). Existing models like the International Metastatic RCC Database Consortium (IMDC) risk model offer limited predictive value for ICI efficacy, as they were primarily developed for patients receiving VEGFR-TKIs (7). Furthermore, tissue-based markers such as PD-L1 expression and tumor mutational burden (TMB) are invasive to obtain and show inconsistent predictive ability in ccRCC (8).

Myeloid-derived suppressor cells (MDSCs), a heterogeneous immunosuppressive cell population present in the TME and peripheral blood, play a critical role in tumor immune evasion and ICI resistance through multiple mechanisms. MDSCs are typically classified into monocytic (M-MDSCs) and polymorphonuclear (PMN-MDSCs) subtypes, each using distinct mechanisms—M-MDSCs primarily through nitric oxide (NO), and PMN-MDSCs via reactive oxygen species (ROS)—to suppress T-cell activity (9–11). MDSCs could also promote tumor angiogenesis by releasing VEGFA and MMP-9 (12, 13). Furthermore, MDSCs interact with other TME components to create an immunosuppressive environment, such as promoting Treg proliferation through CD40, which leads to ICI therapy resistance (14). Studies in other malignancies have linked high circulating MDSC levels with poor prognosis and ICI resistance. In gastric cancer, non-small cell lung cancer (NSCLC), and melanoma, high level of MDSCs was significantly associated with worse prognosis (15, 16). In NSCLC, higher circulating M-MDSC and PMN-MDSC levels were linked to ICIs resistance (17, 18). In ccRCC, the association between circulating MDSC subtypes and ICI treatment response has not been clearly established.

Given that circulating MDSCs can partially reflect their infiltration in the TME and are accessible via minimally invasive blood sampling, they offer a promising use for identifying patients suitable for ICI therapy, enabling precision treatment, and allowing real-time evaluation of treatment efficacy. However, the predictive value of MDSCs for prognosis and ICI response varies significantly across cancer types. In ccRCC, the association between circulating MDSC subtypes and ICI treatment response has not been well established.

This study aimed to investigate whether baseline levels and dynamic changes in total MDSCs, M-MDSCs, and PMN-MDSCs are associated with ICI efficacy in ccRCC patients. By integrating flow cytometry-based quantification of MDSCs with clinical and laboratory parameters in a prospective cohort, we sought to identify potential blood-based biomarkers for predicting ICI response, which may guide personalized immunotherapy strategies in ccRCC.

## 2 Material and methods

### 2.1 Patients and data collection

Patients with ccRCC treated with ICIs at the Department of Urology, Juntendo University between October 2020 and July 2024 who consented to participate in this study were included. The inclusion criteria were: (1) pathological diagnosis of ccRCC; (2) no other primary malignancies; (3) received ICIs for more than three months (including Ipilimumab + Nivolumab, Nivolumab alone after TKI intolerance, or ICI + TKI); and (4) complete MDSCs data from peripheral blood mononuclear cell before and after ICIs treatment. Patients were excluded if they had another primary cancer, received only TKI treatment, or had missing MDSC data. In addition to MDSC measurements, we also collected laboratory data before and after treatment, including uric acid (UA), triglycerides (TG), C-reactive protein (CRP), alkaline phosphatase (ALP), white blood cell count (WBC), neutrophils, lymphocytes, neutrophil-to-lymphocyte ratio (NLR), platelets (PLT), hemoglobin (Hb), lactate dehydrogenase (LDH), total protein (TP), albumin (Alb), calcium, creatinine (SCr), blood urea nitrogen (BUN), and thyroid-stimulating hormone (TSH). Treatment response was assessed based on RECIST 1.1 criteria using the best response recorded between the pre- and post-treatment blood sampling time points. Due to variations in patient clinic visit schedules, post-treatment MDSC sampling was conducted 3 to 9 months after initiating ICI therapy. Patients with complete or partial response (CR or PR) were defined as responders; those with stable or progressive disease (SD or PD) were defined as non-responders.

### 2.2 MDSCs measurement

**Figure 1:**
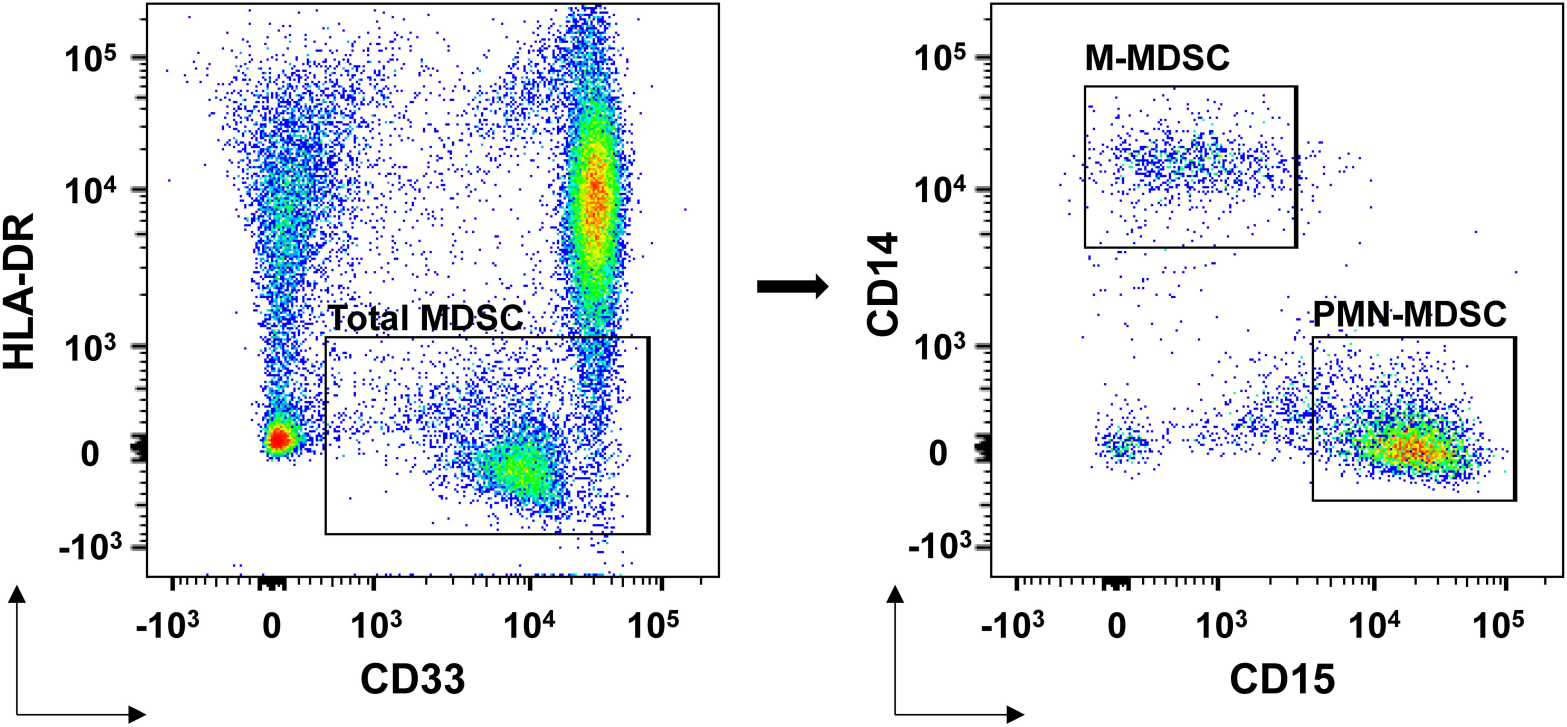
MDSCs flow cytometry identification gating strategies. Total MDSCs were defined as HLA-DR^low/-^ CD33^+^ cells. Within this population, PMN-MDSCs were further defined as HLA-DR^low/-^ CD33^+^ CD15^+^ CD14^-^ cells, and M-MDSCs were identified as HLA-DR^low/-^ CD33^+^ CD15^-^ CD14^+^ cells. All subsets were analyzed as percentages of the total peripheral blood mononuclear cell population. The gating strategy was applied following exclusion of doublets and dead cells.

MDSCs were detected from fresh peripheral blood mononuclear cells (PBMCs) using density gradient centrifugation with Histopaque^®^-1077 (Sigma-Aldrich, St. Louis, MO, USA). 1 × 10^6^ single cells were suspended in 100 μL PBS and incubated for 15 minutes with FcR blocking reagent (BioLegend, San Diego, CA, USA) at room temperature. Fluorescently conjugated antibodies, including HLA-DR-PE-Texas Red, CD14-PerCP-Cy5.5, CD33-PE-Cy7 and CD15-APC-Cy7 (BioLegend, San Diego, CA, USA), were added at appropriate concentrations and incubated at 4°C for 15 minutes, followed by two washes and resuspension of the stained cells in 500 μL buffer containing DAPI (1 μg/mL). Flow cytometry data were acquired using a BD^®^ LSR II flow cytometer (BD Biosciences, San Jose, CA, USA) and analyzed with BD FACSDiva™ software. Further analysis was performed using FlowJo software (Tree Star Inc., Ashland, OR, USA). The gating strategy for MDSC identification was shown in **Figure 1**. The characteristics of total MDSCs, PMN-MDSCs, and M-MDSCs were defined as follows: total MDSCs (HLA-DR^low/-^ CD33^+^), PMN-MDSCs (HLA-DR^low/-^ CD33^+^ CD15^+^ CD14^-^), and M-MDSCs (HLA-DR^low/-^ CD33^+^ CD15^-^ CD14^+^). For each sample, 30,000 PBMC events were collected for flow cytometry analysis to ensure comparability across samples, and MDSC levels were ultimately reported as a percentage of PBMCs.

### 2.3 Statistical analysis

We compared baseline characteristics between ICI responders and non-responders, including age, BMI, smoking and alcohol use, tumor stage, TNM classification, treatment regimens, surgical history, IMDC risk, and comorbidities. Continuous variables were analyzed using Student’s t-test and the Wilcoxon rank-sum test otherwise according to their distribution. Categorical variables were analyzed using Fisher’s exact test.

We further compared MDSC levels (total, M-MDSC, PMN-MDSC), blood counts, and metabolic indicators before and after treatment, as well as their changes during therapy. Missing values (≤20%) were imputed using the random forest method (missForest), and categorical variables were converted to dummy variables.

To assess predictive value, we performed ROC analysis and ranked variables by AUC. To further explore the associations between MDSC-related indicators and clinical parameters, both Pearson correlation and Mantel tests were conducted. Pearson correlation coefficients were calculated to assess linear relationships among all variables. Mantel tests were applied specifically to evaluate matrix-level associations between baseline and change values of MDSC related indicators and other clinical parameters, based on Euclidean distance matrices. Elastic net regression was applied to variables with AUC > 0.6, with standardization and appropriate transformations. Key predictors were selected at λ.1se and validated using Wilcoxon signed-rank and Mann-Whitney U tests.

Finally, univariate and multivariate logistic regression (adjusted for age, disease stage, and treatment regimen) confirmed independent predictors. Differences were considered statistically significant at p values of < 0.05. Statistical analyses were conducted using the R language, version 4.3.4 (R Foundation for Statistical Computing, Vienna, Austria).

## 3 Results

### 3.1 Study population and pre-treatment characteristics

**Figure 2:**
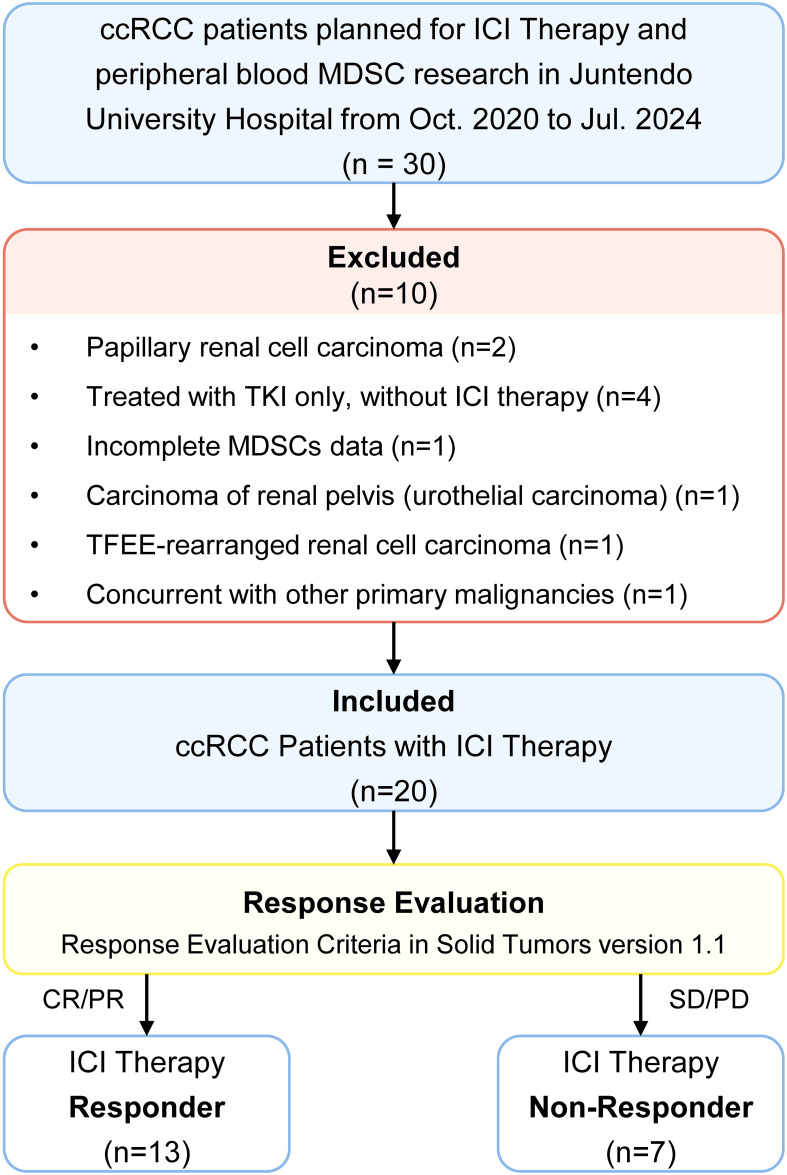
Study population selection strategies. ccRCC, clear cell renal cell carcinoma; ICI therapy, immune checkpoint inhibitor therapy; TKI, tyrosine kinase inhibitor; CR, complete response; PR, partial response; SD, stable disease; PD, progressive disease. MDSC, myeloid-derived suppressor cell; M-MDSC, monocytic myeloid-derived suppressor cell; PMN-MDSC, polymorphonuclear myeloid-derived suppressor cell.

A total of 30 patients scheduled to receive ICI therapy for ccRCC were screened (**Figure 2**). Ten were excluded due to the following reasons: non-ccRCC histology (n = 4), presence of another primary malignancy (n = 1), TKI monotherapy (n = 4), and missing MDSC data (n = 1). Ultimately, 20 patients met all criteria and were enrolled in the study.

Based on RECIST 1.1, 13 patients achieved complete or partial response (CR/PR) were classified as ICI responders, while 7 with stable or progressive disease (SD/PD) were classified as non-responders. Response evaluation was based on the best outcome recorded between the two MDSC sampling timepoints.

Baseline characteristics, including age, sex, BMI, smoking and alcohol use, comorbidities (hypertension, diabetes), tumor stage (at diagnosis and before ICI treatment), T stage, lymph node involvement, distant metastasis, IMDC risk category, and treatment regimens, were comparable between responders and non-responders. No statistically significant differences were observed in any of these variables (all p > 0.05) (**Table 1**).

**Table 1:** Pre-treatment characteristics of ccRCC patients treated with ICI therapy.

Variables	ICI responder (n = 13)	ICI Non-responder (n = 7)	P
Age (y), Mean ± SD	69.54 ± 6.67	68.86 ± 3.63	0.771
Sex, n (%)			1
Female	3 (23)	2 (29)	
Male	10 (77)	5 (71)	
BMI (kg/m^2^), Mean ± SD	23.82 ± 2.68	22.08 ± 3.89	0.316
Smoking, n (%)			0.29
Never	2 (15)	3 (43)	
Current or Former Smoker	11 (85)	4 (57)	
Drinking, n (%)			0.613
Never	10 (77)	4 (57)	
Current or Former Drinker	3 (23)	3 (43)	
Hypertension, n (%)			0.356
No	4 (31)	4 (57)	
Yes	9 (69)	3 (43)	
Diabetes, n (%)			0.521
No	10 (77)	7 (100)	
Yes	3 (23)	0 (0)	
Stage at first diagnosis, n (%)			0.174
Stage 1 & 2	3 (23)	4 (57)	
Stage 3 & 4	10 (77)	3 (43)	
Tumor at first diagnosis, n (%)			0.245
T1	4 (31)	5 (71)	
T2	5 (38)	0 (0)	
T3	3 (23)	2 (29)	
T4	1 (8)	0 (0)	
Lymph node at first diagnosis, n (%)			0.249
N0	9 (69)	7 (100)	
N1	4 (31)	0 (0)	
Metastasis at first diagnosis, n (%)			0.329
M0	7 (54)	6 (86)	
M1	6 (46)	1 (14)	
IMDC risk, n (%)			1
Favorable risk	5 (38)	2 (29)	
Intermediate or poor risk	8 (62)	5 (71)	
Regimen, n (%)			0.35
ICIs (IPI + NIVO/NIVO monotherapy as second line)	5 (38)	5 (71)	
ICI + TKI	8 (62)	2 (29)	
Tumor resection surgery, n (%)			0.158
No	7 (54)	1 (14)	
Yes	6 (46)	6 (86)	
Stage before ICI, n (%)			1
Stage 3	3 (23)	1 (14)	
Stage 4	10 (77)	6 (86)	

BMI, body mass index; ICI, immune checkpoint inhibitor; IMDC risk, international metastatic RCC database consortium risk model; TKI, tyrosine kinase inhibitor.

### 3.2 Comparison of MDSC and other clinical indicators between ICI responders and non-responders: pre-treatment, post-treatment, and changes during treatment

**Figure 3:**
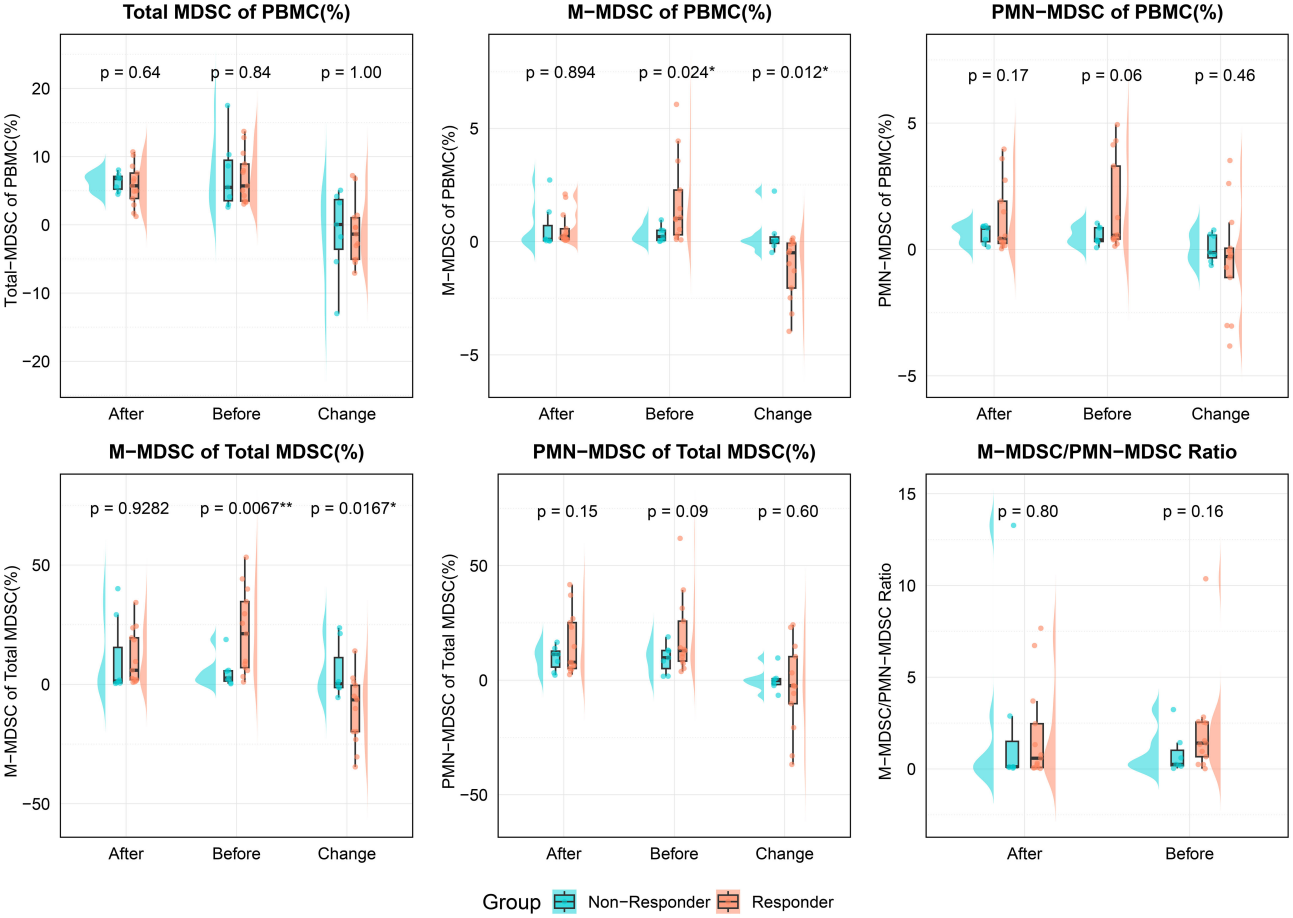
Comparison of MDSC-related indicators between responders and non-responders at different timepoints (before treatment, after treatment, and change during treatment). The variables analyzed included total MDSC of PBMC, M-MDSC and PMN-MDSC of PBMC, M-MDSC and PMN-MDSC of total MDSCs, and the M-MDSC/PMN-MDSC ratio. All values are expressed as percentages of PBMCs, except for the M-MDSC/PMN-MDSC ratio. Statistical significance was assessed using unpaired t-tests; and asterisks denote significance (*p < 0.05, **p < 0.01). PBMC, Peripheral blood mononuclear cell; MDSC, myeloid-derived suppressor cell; M-MDSC, monocytic myeloid-derived suppressor cell; PMN-MDSC, polymorphonuclear myeloid-derived suppressor cell.

In patients receiving ICI therapy for at least 3 months, we compared MDSC levels and other clinical indicators between responders and non-responders at pre-treatment, post-treatment, and for changes during therapy (**Tables 2**, **3**, **Figure 3**).

**Table 2:** Comparison of variables between ICI non-responders and ICI responders before and after ICI treatment.

Variables	ICI responder (n = 13)	ICI Non-responder (n = 7)	P
Total MDSC of PBMC Before (%)	5.71 (3.48, 8.91)	5.5 (3.54, 9.47)	0.817
Total MDSC of PBMC After (%)	5.72 (3.86, 7.6)	6.78 (5.23, 7.11)	0.637
M-MDSC of PBMC Before (%)	1.02 (0.3, 2.28)	0.22 (0.06, 0.5)	0.019*
M-MDSC of PBMC After (%)	0.23 (0.1, 0.57)	0.1 (0.04, 0.71)	0.284
PMN-MDSC of PBMC Before (%)	0.56 (0.41, 3.3)	0.38 (0.32, 0.86)	0.25
PMN-MDSC of PBMC After (%)	0.43 (0.24, 1.91)	0.84 (0.31, 0.92)	0.817
M-MDSC of Total MDSC Before (%)	21.23 (7, 34.7)	2.63 (1.37, 5.7)	0.008*
M-MDSC of Total MDSC After (%)	5.92 (1.97, 19.5)	1.37 (0.62, 15.5)	0.241
PMN-MDSC of Total MDSC Before (%)	12.93 (8.33, 25.8)	9.8 (5.08, 12.95)	0.241
PMN-MDSC of Total MDSC After (%)	7.8 (5.1, 25.13)	11.32 (5.7, 12.67)	0.536
M-MDSC/PMN-MDSC Before	1.41 (0.67, 2.55)	0.25 (0.18, 1.02)	0.183
M-MDSC/PMN-MDSC After	0.59 (0.08, 2.47)	0.12 (0.1, 1.5)	0.699
UA Before (mg/dL)	6.46 ± 1.91	6.44 ± 1.66	0.982
UA After (mg/dL)	6.52 ± 1.28	5.57 ± 1.57	0.204
TG Before (mg/dL)	106.5 (81.75, 170.5)	108.5 (95, 215.75)	0.708
TG After (mg/dL)	159 (113.5, 277.5)	123 (108, 176)	0.328
CRP Before (mg/dL)	0.33 (0.25, 1.69)	0.28 (0.17, 0.36)	0.475
CRP After (mg/dL)	0.47 (0.15, 0.69)	0.19 (0.08, 0.52)	0.267
WBC Before (10^9^/L)	6.4 (5.6, 9.9)	5.7 (4.9, 6.4)	0.25
WBC After (10^9^/L)	6.70 (5.3, 8.4)	7.60 (7.20, 9.05)	0.544
Neutrophil Count Before (10^9^/L)	4.53 (3.33, 6.11)	3.13 (2.84, 3.68)	0.03*
Neutrophil Count After (10^9^/L)	4.35 (2.90, 5.37)	4.04 (4.00, 4.40)	0.979
Lymphocyte Count Before (10^9^/L)	1.61 ± 0.52	1.65 ± 0.42	0.837
Lymphocyte Count After (10^9^/L)	1.83 ± 0.76	1.95 ± 0.76	0.771
NLR Before	3.1 (2.29, 3.71)	2.1 (1.73, 2.28)	0.03*
NLR After	2.28 (1.91, 3.02)	2.37 (1.56, 2.57)	1
Platelet Before (10^9^/L)	237 (172, 316)	304 (264, 349)	0.328
Platelet After (10^9^/L)	190 (161, 216)	266 (260.5, 312.5)	0.03*
Hb Before (g/dL)	12.55 ± 2.21	12.01 ± 1.73	0.556
Hb After (g/dL)	13.42 ± 1.77	12.36 ± 2.31	0.316
ALP Before (IFCC, U/L)	95 (78, 117)	113 (99, 174)	0.234
ALP After (IFCC, U/L)	71 (67, 96)	80 (72.25, 89.25)	0.568
LDH Before (IFCC, U/L)	191.23 ± 48.39	201 ± 33.42	0.603
LDH After (IFCC, U/L)	212.31 ± 53.09	214.29 ± 62.11	0.968
TP Before (g/dL)	7.2 ± 0.58	6.74 ± 0.68	0.159
TP After (g/dL)	6.9 ± 0.44	6.59 ± 1.18	0.52
Alb Before (g/dL)	3.95 ± 0.42	3.8 ± 0.51	0.532
Alb After (g/dL)	3.85 ± 0.32	3.61 ± 0.91	0.841
Calcium Before (mg/dL)	9.2 (9.1, 9.6)	9.1 (8.65, 9.35)	0.358
Calcium After (mg/dL)	9.4 (9.1, 9.6)	9.2 (8.65, 9.5)	0.212
SCr Before (mg/dL)	0.94 (0.81, 1.08)	1 (0.87, 1.39)	0.817
SCr After (mg/dL)	1.01 (0.85, 1.1)	1.03 (0.85, 1.12)	0.937
BUN Before (mg/dL)	22.46 ± 10.92	17.96 ± 8.58	0.781
BUN After (mg/dL)	18.92 ± 4.05	15.14 ± 6.2	0.181
TSH Before (mIU/L)	2.86 (1.43, 3.83)	2.13 (1.07, 3.66)	0.663
TSH After (mIU/L)	2.79 (2.16, 6.37)	5.75 (4.24, 7.46)	0.241

Normally distributed numerical variables were presented as mean ± SD. Non-normally distributed numerical variables were presented as median (Q1, Q3). MDSC, myeloid-derived suppressor cell; M-MDSC, monocytic myeloid-derived suppressor cell; PMN-MDSC, polymorphonuclear myeloid-derived suppressor cell; UA, blood uric acid; TG, triglyceride; CRP, C-reactive protein; ALP, alkaline phosphatase; WBC, white blood cell; NLR, neutrophil-to-lymphocyte ratio; Hb, hemoglobin; LDH, lactate dehydrogenase; TP, total protein; Alb, albumin; SCr, serum creatinine; BUN, blood urea nitrogen; TSH, thyroid stimulating hormone.

Bold values and asterisks* denote statistical significance at p < 0.05.

**Table 3:** Comparison of changes in variables between ICI non-responders and ICI responders during ICI treatment.

Variables	ICI responder (n = 13)	ICI Non-responder (n = 7)	P
Total MDSC Change (%)	-1.1 ± 4.44	-1.09 ± 6.41	0.999
M-MDSC of PBMC Change (%)	-0.49 (-2.05, -0.07)	0.03 (-0.08, 0.21)	0.024*
PMN-MDSC of PBMC Change (%)	-0.28 (-1.11, 0.05)	-0.12 (-0.33, 0.56)	0.451
M-MDSC of Total MDSC Change (%)	-10.6 ± 14.08	5.47 ± 11.84	0.017*
PMN-MDSC of Total MDSC Change (%)	-2.96 ± 19.07	0.05 ± 4.93	0.599
UA Change (mg/dL)	-0.13 ± 0.96	-0.87 ± 0.86	0.107
TG Change (mg/dL)	43.08 ± 105.54	-77.25 ± 163.06	0.241
CRP Change (mg/dL)	-0.01 (-0.07, 0.48)	0.01 (-0.22, 0.06)	0.526
WBC Change (10^9^/L)	-0.5 ± 2.9	1.69 ± 3.15	0.155
Neutrophil Count Change (10^9^/L)	-0.9 (-1.21, 0.36)	1.07 (0.53, 1.35)	0.029*
Lymphocyte Count Change (10^9^/L)	0.23 ± 0.6	0.18 ± 0.69	0.89
NLR Change	-0.43 (-1.51, 0.13)	0.11 (-0.05, 0.76)	0.127
Platelet Change (10^9^/L)	-42.92 ± 63.92	4.29 ± 72.79	0.177
Hb Change (g/dL)	0.86 ± 2.02	0.34 ± 2.46	0.642
ALP Change (IFCC, U/L)	-7 (-27, 1)	-19.5 (-89, -8.5)	0.368
LDH Change (IFCC, U/L)	21.08 ± 59.61	13.29 ± 78.61	0.823
TP Change (g/dL)	-0.3 ± 0.47	-0.16 ± 0.94	0.716
Alb Change (g/dL)	-0.1 ± 0.39	-0.19 ± 0.58	0.734
Calcium Change (mg/dL)	0.02 ± 0.73	-0.07 ± 0.81	0.801
SCr Change (mg/dL)	0.01 (-0.03, 0.21)	-0.18 (-0.3, -0.02)	0.088
BUN Change (mg/dL)	0 (-2, 3)	-3 (-7.5, -1.5)	0.266
TSH Change (mIU/L)	0.08 ± 3.12	2.92 ± 3.12	0.075

Normally distributed numerical variables were presented as mean ± SD. Non-normally distributed numerical variables were presented as median (Q1, Q3). MDSC, myeloid-derived suppressor cell; M-MDSC, monocytic myeloid-derived suppressor cell; PMN-MDSC, polymorphonuclear myeloid-derived suppressor cell; UA, blood uric acid; TG, triglyceride; CRP, C-reactive protein; ALP, alkaline phosphatase; WBC, white blood cell; NLR, neutrophil-to-lymphocyte ratio; Hb, hemoglobin; LDH, lactate dehydrogenase; TP, total protein; Alb, albumin; SCr, serum creatinine; BUN, blood urea nitrogen; TSH, thyroid stimulating hormone.

Bold values and asterisks* denote statistical significance at p < 0.05.

Total MDSC levels (as % of PBMCs) showed no significant differences between groups at any time point. However, responders had significantly higher pre-treatment M-MDSC levels (1.02% vs. 0.22%, p = 0.019) and a greater proportion of M-MDSCs within total MDSCs (21.23% vs. 2.63%, p = 0.008). These differences disappeared post-treatment. PMN-MDSC levels and their proportions showed no significant differences at any time point.

During treatment, responders showed a significant reduction in both M-MDSC levels (-0.49% vs. 0.03%, p = 0.024) and their proportion within total MDSCs (-10.6% vs. 5.47%, p = 0.017). No significant differences were observed in PMN-MDSC or total MDSC changes between groups.

Among other clinical markers, responders had higher pre-treatment neutrophil counts and NLR (p = 0.03 for both). Post-treatment, platelet levels were lower in responders (p = 0.03), and during treatment, neutrophil counts decreased in responders but increased in non-responders (p = 0.029). These results suggest that M-MDSC levels, especially their baseline levels and treatment-related decline, may be valuable in predicting ICI response in ccRCC patients.

### 3.3 AUC and correlation of variables

**Figure 4:**
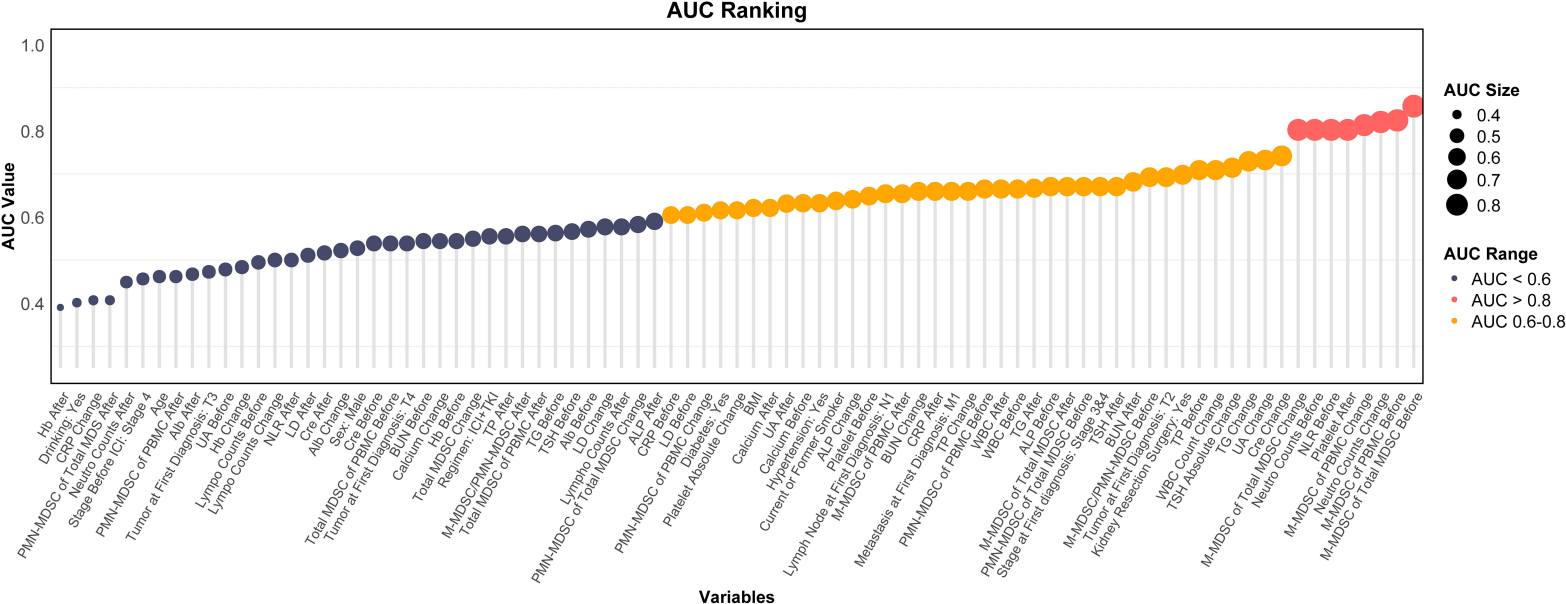
Ranking plot of variables by AUC for predicting ICI response. AUC, area under the curve; MDSC, myeloid-derived suppressor cell; M-MDSC, monocytic myeloid-derived suppressor cell; PMN-MDSC, polymorphonuclear myeloid-derived suppressor cell; UA, blood uric acid; TG, triglyceride; CRP, C-reactive protein; ALP, alkaline phosphatase; WBC, white blood cell; NLR, neutrophil-to-lymphocyte ratio; Hb, hemoglobin; LDH, lactate dehydrogenase; TP, total protein; Alb, albumin; SCr, serum creatinine; BUN, blood urea nitrogen; TSH, thyroid stimulating hormone; BMI, body mass index.

ROC analysis was conducted for all variables listed in **Tables 1**-**3**. Variables with AUC > 0.8 were primarily related to M-MDSCs, including pre-treatment M-MDSC levels, their proportion within total MDSCs, and their changes during treatment. High AUC values were also observed for pre-treatment NLR, neutrophil count, and post-treatment platelet levels. Variables with AUC between 0.6 and 0.8 were mostly related to changes in metabolic markers such as creatinine, triglycerides, and uric acid. Variables with AUC < 0.6 were considered to have limited predictive value (**Figure 4**).

**Figure 5:**
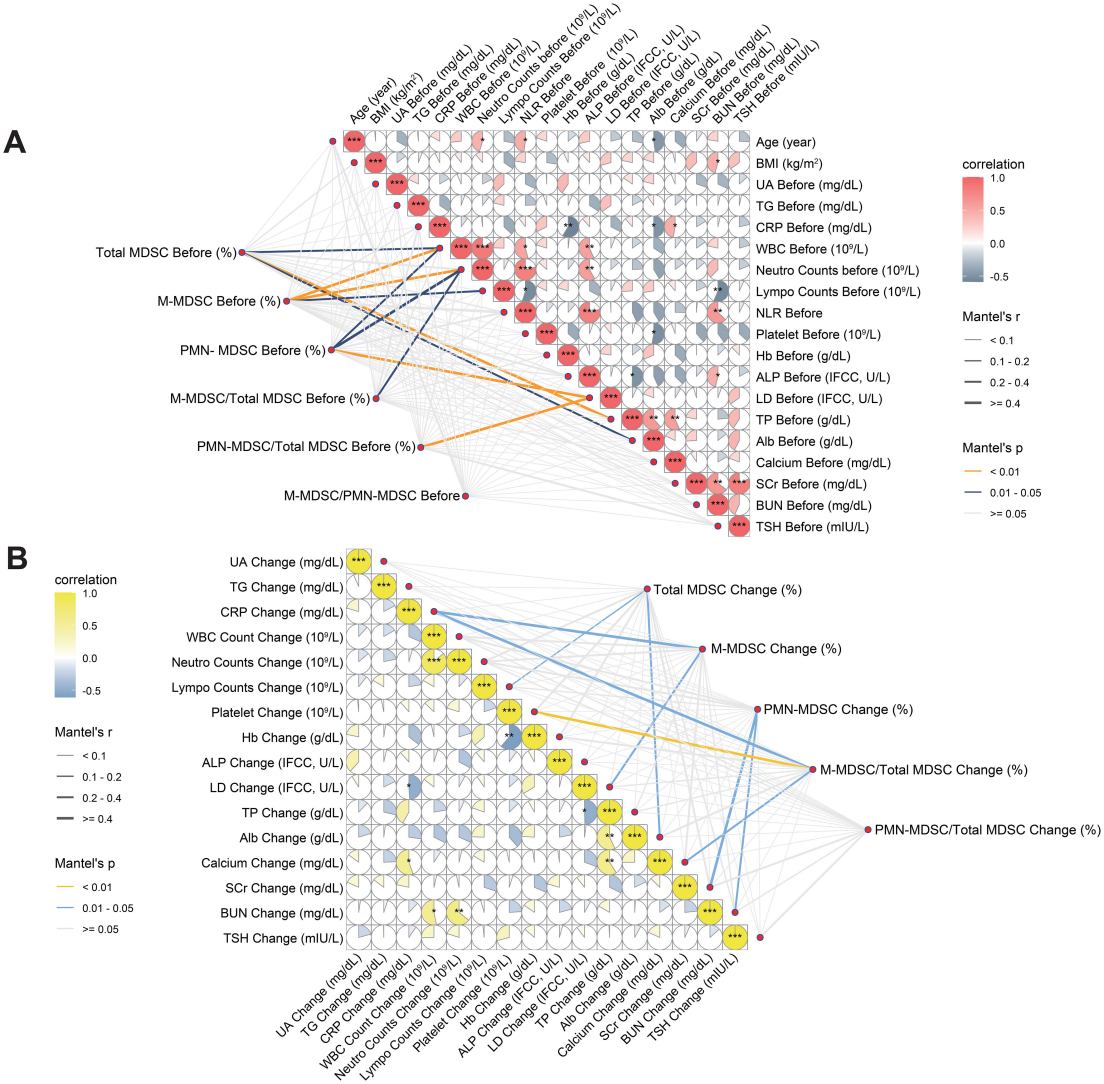
Correlation analyses between MDSC-related variables and clinical parameters at baseline and during treatment. **(A)** Associations between baseline MDSC indicators and pre-treatment clinical parameters. **(B)** Associations between changes in MDSC indicators and concurrent changes in clinical parameters. Both Pearson correlation coefficients (color scale) and Mantel test results (network lines) are shown. Circle color intensity indicates Pearson r values; asterisks mark levels of statistical significance (*p < 0.05, **p < 0.01, ***p < 0.001). Mantel’s r is represented by edge thickness, and p-values by edge color (yellow: p < 0.01; blue: p = 0.01–0.05; grey: p ≥ 0.05). MDSC, myeloid-derived suppressor cell; M-MDSC, monocytic myeloid-derived suppressor cell; PMN-MDSC, polymorphonuclear myeloid-derived suppressor cell; UA, blood uric acid; TG, triglyceride; CRP, C-reactive protein; ALP, alkaline phosphatase; WBC, white blood cell; NLR, neutrophil-to-lymphocyte ratio; Hb, hemoglobin; LDH, lactate dehydrogenase; TP, total protein; Alb, albumin; SCr, serum creatinine; BUN, blood urea nitrogen; TSH, thyroid stimulating hormone; BMI, body mass index.

Pearson correlation analysis revealed strong associations among MDSC-related variables, particularly between pre-treatment M-MDSC levels and changes during treatment. These variables showed only weak correlations (r < 0.5) with most clinical indicators. However, moderate positive correlations were observed with WBC count, NLR, and neutrophils, while moderate negative correlations were found with total protein, albumin, and calcium (**Supplementary Figure S1**). Mantel test revealed that pre-treatment MDSC-related indicators exhibited moderate matrix-level correlations (Mantel’s *r* = 0.2–0.4, p < 0.05) with immune-related parameters such as baseline WBC and lymphocyte counts, as well as liver function markers including LDH, albumin, and total protein levels (**Figure 5A**, **Supplementary Table S1**). Changes in MDSC-related indicators demonstrated significant Mantel correlations with alterations in metabolic parameters, including LDH, creatinine, calcium, and platelet counts (p < 0.05; **Figure 5B**, **Supplementary Table S2**). In contrast to baseline MDSC profiles, dynamic changes in MDSCs during treatment exhibited no significant associations with immune-related parameters, suggesting that MDSC kinetics may reflect distinct biological mechanisms and hold potential as independent biomarkers of therapeutic response.

### 3.4 Elastic net regression analysis for key variables of ICI response selection

**Figure 6:**
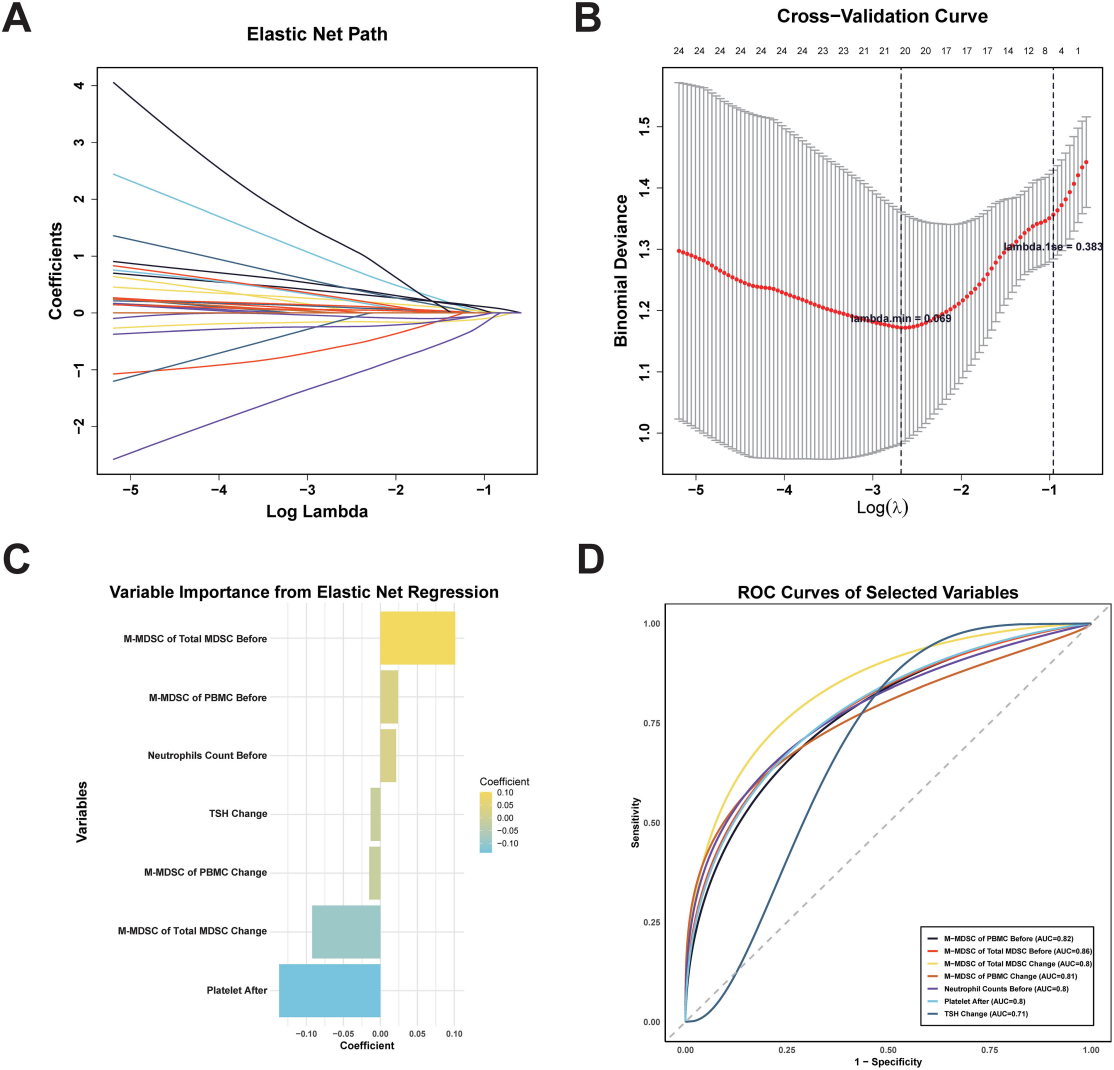
Elastic net regression for feature selection and ROC Curves of selected variables for predicting ICI treatment response. **(A)** Elastic net regression path. **(B)** Cross-validation graph. λ.min (minimum cross-validation error) and λ.1se (most conservative within one standard error). **(C)** Regression coefficients rankings of the seven variables with non-zero coefficients selected at λ.1se. **(D)** ROC curves of seven selected variables for predicting ICI therapy response. MDSC, myeloid-derived suppressor cell; M-MDSC, monocytic myeloid-derived suppressor cell; PMN-MDSC, polymorphonuclear myeloid-derived suppressor cell; TSH, thyroid stimulating hormone.

We applied elastic net regression to 47 variables with AUC > 0.6 (from **Figure 4**) to identify predictors of ICI response (**Figure 6A**). Using cross-validation, λ.1se (0.383) was selected to balance model simplicity and performance (**Figure 6B**). Seven variables with non-zero coefficients were selected (**Figure 6C**): pre-treatment M-MDSC proportion within total MDSCs, pre-treatment M-MDSC level, pre-treatment neutrophil count, changes in M-MDSC level and proportion during treatment, changes in TSH, and post-treatment platelet count. The pre-treatment M-MDSC proportion had the highest coefficient, indicating its strong predictive contribution. ROC analysis showed that all selected variables had AUC > 0.7 (**Figure 6D**), with pre-treatment M-MDSC proportion and level showing the highest AUC values (0.86 and 0.82, respectively). These findings suggest that M-MDSC–related indicators, particularly their pre-treatment levels and proportions, may serve as reliable predictors of ICI response in ccRCC.

### 3.5 Statistical tests and logistic regression analysis of selected variables

**Figure 7:**
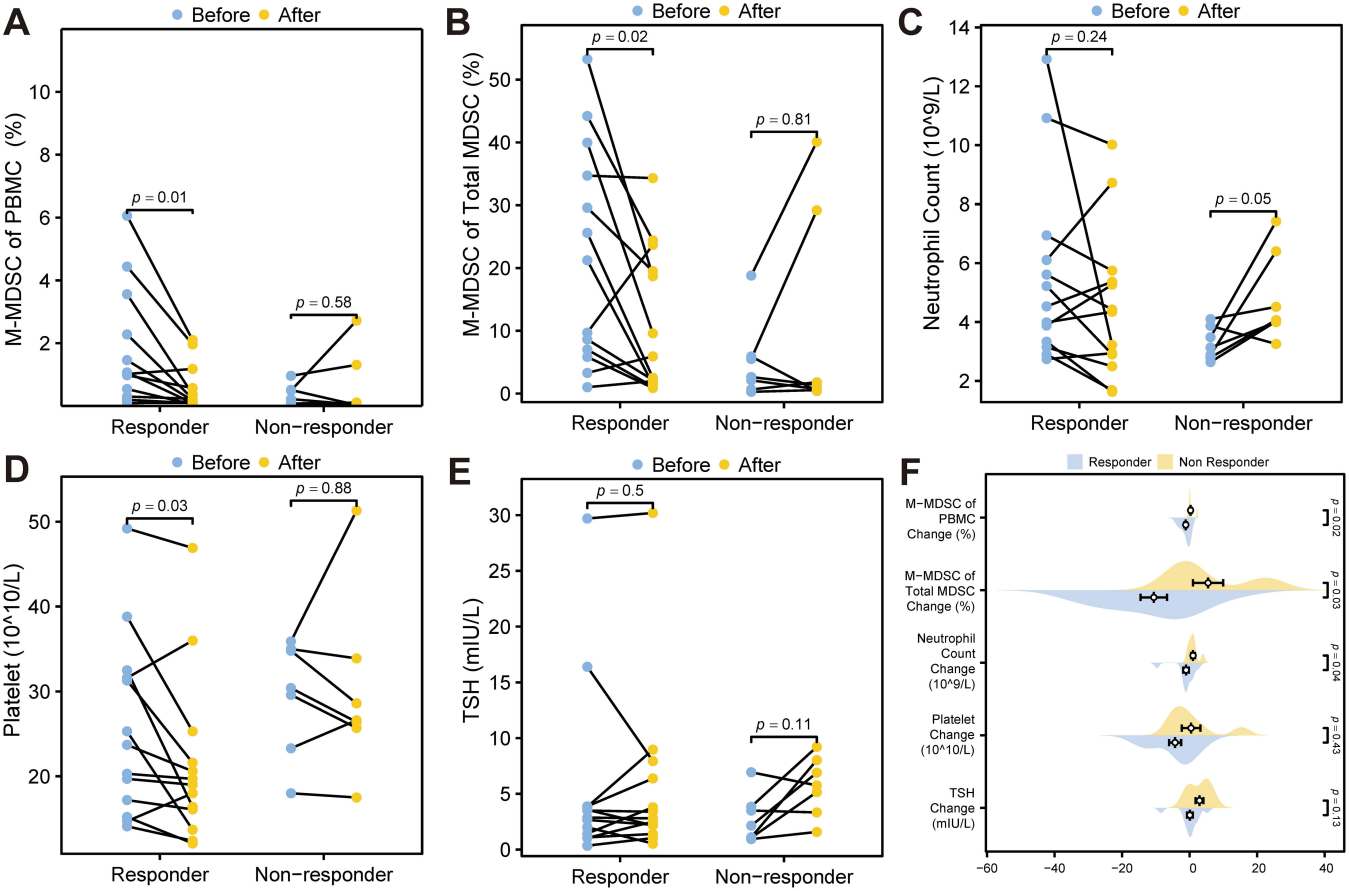
Statistical tests of potential predictors. **(A)** Wilcoxon signed rank test for M-MDSC levels before and after ICI treatment. **(B)** Wilcoxon signed rank test of the proportion of M-MDSC within total MDSCs before and after ICI treatment. **(C)** Wilcoxon signed rank test for neutrophil count levels before and after treatment. **(D)** Wilcoxon signed rank test for platelet levels before and after treatment. **(E)** Wilcoxon signed rank test for TSH levels before and after treatment. **(F)** Mann-Whitney U test comparing changes in TSH, platelet levels, neutrophil count, M-MDSC proportion within total MDSCs, and M-MDSC of PBMC during ICI treatment between ICI responders and non-responders. MDSC, myeloid-derived suppressor cell; M-MDSC, monocytic myeloid-derived suppressor cell; TSH, thyroid stimulating hormone.

Wilcoxon signed rank tests showed significant reductions in M-MDSC levels (p = 0.01, **Figure 7A**), M-MDSC proportion within total MDSCs (p = 0.02, **Figure 7B**), and platelet counts (p = 0.03, **Figure 7D**) in ICI responders after treatment. These changes were not observed in non-responders (all p > 0.05). Neutrophil counts increased marginally in non-responders (p = 0.05, **Figure 7C**), while TSH levels remained unchanged in both groups (**Figure 7E**). Mann-Whitney U tests comparing changes between groups yielded consistent results (**Figure 7F**).

Univariate logistic regression indicated that pre-treatment M-MDSC proportion (OR = 2.787, 95% CI: 1.069–7.263, p = 0.036) and M-MDSC levels (OR = 2.217, 95% CI: 1.007–4.884, p = 0.048) were significantly associated with ICI response. Changes in M-MDSC proportion (p = 0.061) and TSH (p = 0.078) showed borderline significance (**Table 4**). Multivariate logistic regression, adjusted for age, disease stage, and treatment regimen, confirmed that both pre-treatment M-MDSC proportion (Adjusted OR = 5.764, 95% CI: 1.125–29.527, p = 0.036) and M-MDSC level (Adjusted OR = 3.082, 95% CI: 1.047–9.075, p = 0.041) were independent predictors of ICI response (**Table 5**).

**Table 4:** Univariate logistic regression analysis of variables associated with treatment response.

Variable	OR	95% CI (Lower)	95% CI (Upper)	P
M-MDSC of PBMC Before	2.217	1.007	4.884	0.048*
M-MDSC of PBMC Change	0.083	0.003	2.606	0.157
M-MDSC of Total MDSC Before	2.787	1.069	7.263	0.036*
M-MDSC of Total MDSC Change	0.897	0.8	1.005	0.061
Neutrophil Count Before	3.272	0.793	13.498	0.101
Platelet After	0.992	0.981	1.002	0.112
TSH Change	0.686	0.451	1.043	0.078

OR, odds ratio; CI, confidence intervals; MDSC, myeloid-derived suppressor cell; M-MDSC, monocytic myeloid-derived suppressor cell; TSH, thyroid stimulating hormone.

Bold values and asterisks* denote statistical significance at p < 0.05.

**Table 5:** Multivariate logistic regression of variables selected by elastic net regression for ICI response (each variable adjusted for age, pre-treatment stage and treatment regimens).

Variable	Adjusted OR	95% CI (Lower)	95% CI (Upper)	P
M-MDSC of PBMC Before	3.082	1.047	9.075	0.041*
M-MDSC of PBMC Change	0.073	0.001	3.821	0.195
M-MDSC of Total MDSC Before	5.764	1.125	29.527	0.036*
M-MDSC of Total MDSC Change	0.879	0.764	1.012	0.072
Neutrophil Count Before	3.986	0.692	22.962	0.122
Platelet After	0.990	0.977	1.003	0.137
TSH Change	0.597	0.333	1.069	0.083

Adjusted OR, adjusted odds ratio; CI, confidence interval; MDSC, myeloid-derived suppressor cell; M-MDSC, monocytic myeloid-derived suppressor cell; TSH, thyroid stimulating hormone.

Bold values and asterisks* denote statistical significance at p < 0.05.

## 4 Discussion

This study explored the relationship between peripheral MDSC levels and ICI response in ccRCC patients. We identified pre-treatment M-MDSC levels and their proportion within total MDSCs as independent predictors of treatment efficacy, both of which significantly decreased in responders following ICI therapy. These findings highlight the potential role of M-MDSCs as accessible blood-based biomarkers for guiding immunotherapy in ccRCC.

While immunotherapy has changed the treatment landscape for ccRCC, predicting which patients will benefit remains challenging. US-FDA-approved biomarkers such as PD-L1 expression and tumor mutational burden (TMB) showed limitations on invasiveness and inconsistent accuracy (19, 20). Blood-based indicators, such as those in the IMDC risk model, were originally developed for VEGF-TKI-treated patients, but some components—like neutrophil count, NLR, and platelet levels—have also shown potential predictive value for ICI therapy (7, 21). In our study, responders had significantly higher baseline neutrophil counts and NLR, and lower post-treatment platelet levels, consistent with prior findings. However, due to their non-specificity, these markers may not directly reflect tumor–immune interactions.

MDSCs, particularly the M-MDSC subpopulation, have emerged as key immunosuppressive cells in the TME (22). According to prior studies, higher baseline MDSC levels have been associated with worse ICI efficacy in melanoma, prostate cancer, and NSCLC (23, 24). However, the predictive value of MDSC subtypes varies by tumor type (25, 26). A meta-analysis involving 1035 patients treated with ICIs found that lower circulating MDSC levels were associated with improved survival, although heterogeneity existed based on tumor type and MDSC subtype (27). Notably, reductions in M-MDSCs during treatment were also associated with better outcomes in melanoma patients (28).

Contrary to prior studies, we found that higher pre-treatment M-MDSC levels and their proportions within total MDSCs were associated with better ICI response in ccRCC, which may attribute to tumor-specific immune dynamics and differences in the thresholds used to define high MDSC levels across studies (29). For example, M-MDSC cutoff values in melanoma ranged from 0.73% to 7.1% of live PBMCs, while our median in responders was 1.02%. Additionally, circulating MDSCs may differ in function from those in the TME, and their predictive value could vary depending on tumor burden and immune status (30, 31). Furthermore, our study focused on short-term response, while prior research mainly assessed longtime outcomes like PFS and OS.

We propose that higher pre-treatment M-MDSC levels in ccRCC may indicate a more immunologically active state, which facilitates a better response to ICI therapy. MDSC development involves tumor-driven signals and inflammatory cytokines such as IL-17A, TNF-α, and G-CSF (32, 33). Chemokines like *CCL2* and *CCL5* facilitate MDSC infiltrating to tumors (34, 35), while factors such as *STAT3*, *IRF8*, and *HMGB1* promote their immunosuppressive function in the TME (36, 37). In this context, a higher baseline M-MDSC level in ccRCC patients may suggest that the tumor is under immune pressure and has initiated the recruitment of suppressive cells, indicating a TME enriched in T cells that could be responsive to ICI treatment.

Supporting this hypothesis, we observed a significant reduction in peripheral M-MDSC levels and their proportion within total MDSCs after ICI treatment in responders. Previous research has also shown that M-MDSC levels correlate with tumor burden in RCC (38), and in our cohort, non-responders had a notably lower median pre-treatment M-MDSC level (0.22%), which may reflect immune exhaustion or limited baseline immune activation.

This study employed multiple statistical approaches within a prospective cohort design to identify both the baseline circulating M-MDSC level and its proportion within total MDSCs as predictive biomarkers of ICI efficacy. To our knowledge, this is the first study to highlight the predictive value of circulating M-MDSC composition in ccRCC patients treated with ICIs. However, this study has several limitations. Firstly, the limited sample size may restrict the universality of the findings. Secondly, this study only focused on short-term treatment response (CR/PR vs. SD/PD) but did not evaluate long term outcomes, such as PFS or OS. Another potential limitation was the use of elastic net regression for variable selection in a small sample cohort may introduce selection bias, the robustness of the selected features should be interpreted with caution. Additionally, different ICI-based regimens (ICI + ICI, ICI + TKI, ICI after TKI intolerance) could have influenced immune dynamics despite statistical adjustment. Finally, our study was observational and lacked mechanistic exploration.

To further validate the predictive value of M-MDSCs in ICI treatment response, future studies should expand sample sizes and conduct large-scale multicenter studies, and include patients only receiving Ipilimumab + Nivolumab without concomitant TKIs. Functional studies are also required to elucidate the role of M-MDSCs in tumor immune regulation, including assessing PD-1/PD-L1 expression on M-MDSCs, evaluating their immunosuppressive capacity, and analyzing the secretion of immunosuppressive and pro-inflammatory cytokines such as IL-10, IL-6, and TGF-β through single-cell sequencing and flow cytometry (39). Finally, combining M-MDSCs with other immune markers could help develop more accurate ICI response predictive models enabling personalized treatment for ccRCC patients.

## 5 Conclusion

Our study first revealed that pre-treatment M-MDSC levels and the M-MDSC proportion within total MDSCs were independent predictors of ICI treatment response in ccRCC patients. These findings provided potential biomarkers for personalized immunotherapy and insights into tumor immune evasion and treatment mechanisms.

## Data Availability

The original contributions presented in the study are included in the article/**Supplementary Material**. Further inquiries can be directed to the corresponding authors.

## References

[1] Hu J Tan P Ishihara M Bayley NA Schokrpur S Reynoso JG. Tumor heterogeneity in vhl drives metastasis in clear cell renal cell carcinoma. Signal Transduct Target Ther. (2023) 8:155. doi: 10.1038/s41392-023-01362-2, PMID: 37069149 PMC10110583

[2] Vuong L Kotecha RR Voss MH Hakimi AA. Tumor microenvironment dynamics in clear-cell renal cell carcinoma. Cancer Discov. (2019) 9:1349–57. doi: 10.1158/2159-8290.Cd-19-0499, PMID: 31527133 PMC6774890

[3] Ljungberg B Albiges L Abu-Ghanem Y Bedke J Capitanio U Dabestani S. European association of urology guidelines on renal cell carcinoma: the 2022 update. Eur Urol. (2022) 82:399–410. doi: 10.1016/j.eururo.2022.03.006, PMID: 35346519

[4] Motzer RJ Tannir NM McDermott DF Arén Frontera O Melichar B Choueiri TK. Nivolumab plus ipilimumab versus sunitinib in advanced renal-cell carcinoma. N Engl J Med. (2018) 378:1277–90. doi: 10.1056/NEJMoa1712126, PMID: 29562145 PMC5972549

[5] Choueiri TK Motzer RJ Rini BI Haanen J Campbell MT Venugopal B. Updated efficacy results from the javelin renal 101 trial: first-line avelumab plus axitinib versus sunitinib in patients with advanced renal cell carcinoma. Ann Oncol. (2020) 31:1030–9. doi: 10.1016/j.annonc.2020.04.010, PMID: 32339648 PMC8436592

[6] Aggen DH Drake CG Rini BI. Targeting pd-1 or pd-L1 in metastatic kidney cancer: combination therapy in the first-line setting. Clin Cancer Res. (2020) 26:2087–95. doi: 10.1158/1078-0432.Ccr-19-3323, PMID: 31948999

[7] Heng DY Xie W Regan MM Harshman LC Bjarnason GA Vaishampayan UN. External validation and comparison with other models of the international metastatic renal-cell carcinoma database consortium prognostic model: A population-based study. Lancet Oncol. (2013) 14:141–8. doi: 10.1016/s1470-2045(12)70559-4, PMID: 23312463 PMC4144042

[8] Gubin MM Zhang X Schuster H Caron E Ward JP Noguchi T. Checkpoint blockade cancer immunotherapy targets tumour-specific mutant antigens. Nature. (2014) 515:577–81. doi: 10.1038/nature13988, PMID: 25428507 PMC4279952

[9] Nagaraj S Gupta K Pisarev V Kinarsky L Sherman S Kang L. Altered recognition of antigen is a mechanism of cd8+ T cell tolerance in cancer. Nat Med. (2007) 13:828–35. doi: 10.1038/nm1609, PMID: 17603493 PMC2135607

[10] Mazzoni A Bronte V Visintin A Spitzer JH Apolloni E Serafini P. Myeloid suppressor lines inhibit T cell responses by an no-dependent mechanism. J Immunol. (2002) 168:689–95. doi: 10.4049/jimmunol.168.2.689, PMID: 11777962

[11] Wang JC Sun L. Pd-1/pd-L1, mdsc pathways, and checkpoint inhibitor therapy in ph(-) myeloproliferative neoplasm: A review. Int J Mol Sci. (2022) 23:5837. doi: 10.3390/ijms23105837, PMID: 35628647 PMC9143160

[12] Sorrentino C Miele L Porta A Pinto A Morello S. Myeloid-derived suppressor cells contribute to A2b adenosine receptor-induced vegf production and angiogenesis in a mouse melanoma model. Oncotarget. (2015) 6:27478–89. doi: 10.18632/oncotarget.4393, PMID: 26317647 PMC4695003

[13] Varikuti S Oghumu S Elbaz M Volpedo G Ahirwar DK Alarcon PC. Stat1 gene deficient mice develop accelerated breast cancer growth and metastasis which is reduced by il-17 blockade. Oncoimmunology. (2017) 6:e1361088. doi: 10.1080/2162402x.2017.1361088, PMID: 29147627 PMC5674966

[14] Pan PY Ma G Weber KJ Ozao-Choy J Wang G Yin B. Immune stimulatory receptor cd40 is required for T-cell suppression and T regulatory cell activation mediated by myeloid-derived suppressor cells in cancer. Cancer Res. (2010) 70:99–108. doi: 10.1158/0008-5472.Can-09-1882, PMID: 19996287 PMC2805053

[15] Wang L Chang EW Wong SC Ong SM Chong DQ Ling KL. Increased myeloid-derived suppressor cells in gastric cancer correlate with cancer stage and plasma S100a8/A9 proinflammatory proteins. J Immunol. (2013) 190:794–804. doi: 10.4049/jimmunol.1202088, PMID: 23248262

[16] Chevolet I Speeckaert R Schreuer M Neyns B Krysko O Bachert C. Clinical significance of plasmacytoid dendritic cells and myeloid-derived suppressor cells in melanoma. J Transl Med. (2015) 13:9. doi: 10.1186/s12967-014-0376-x, PMID: 25592374 PMC4326397

[17] Limagne E Richard C Thibaudin M Fumet JD Truntzer C Lagrange A. Tim-3/galectin-9 pathway and mmdsc control primary and secondary resistances to pd-1 blockade in lung cancer patients. Oncoimmunology. (2019) 8:e1564505. doi: 10.1080/2162402x.2018.1564505, PMID: 30906658 PMC6422400

[18] Passaro A Mancuso P Gandini S Spitaleri G Labanca V Guerini-Rocco E. Gr-mdsc-linked asset as a potential immune biomarker in pretreated nsclc receiving nivolumab as second-line therapy. Clin Transl Oncol. (2020) 22:603–11. doi: 10.1007/s12094-019-02166-z, PMID: 31254252

[19] Havel JJ Chowell D Chan TA. The evolving landscape of biomarkers for checkpoint inhibitor immunotherapy. Nat Rev Cancer. (2019) 19:133–50. doi: 10.1038/s41568-019-0116-x, PMID: 30755690 PMC6705396

[20] Chowell D Yoo SK Valero C Pastore A Krishna C Lee M. Improved prediction of immune checkpoint blockade efficacy across multiple cancer types. Nat Biotechnol. (2022) 40:499–506. doi: 10.1038/s41587-021-01070-8, PMID: 34725502 PMC9363980

[21] Young M Tapia JC Szabados B Jovaisaite A Jackson-Spence F Nally E. Nlr outperforms low hemoglobin and high platelet count as predictive and prognostic biomarker in metastatic renal cell carcinoma treated with immune checkpoint inhibitors. Clin Genitourin Cancer. (2024) 22:102072. doi: 10.1016/j.clgc.2024.102072, PMID: 38615487

[22] Hernandez C Arasanz H Chocarro L Bocanegra A Zuazo M Fernandez-Hinojal G. Systemic blood immune cell populations as biomarkers for the outcome of immune checkpoint inhibitor therapies. Int J Mol Sci. (2020) 21:2411. doi: 10.3390/ijms21072411, PMID: 32244396 PMC7177687

[23] Meyer C Cagnon L Costa-Nunes CM Baumgaertner P Montandon N Leyvraz L. Frequencies of circulating mdsc correlate with clinical outcome of melanoma patients treated with ipilimumab. Cancer Immunol Immunother. (2014) 63:247–57. doi: 10.1007/s00262-013-1508-5, PMID: 24357148 PMC11029062

[24] Santegoets SJ Stam AG Lougheed SM Gall H Jooss K Sacks N. Myeloid derived suppressor and dendritic cell subsets are related to clinical outcome in prostate cancer patients treated with prostate gvax and ipilimumab. J Immunother Cancer. (2014) 2:31. doi: 10.1186/s40425-014-0031-3, PMID: 26196012 PMC4507359

[25] Hou A Hou K Huang Q Lei Y Chen W. Targeting myeloid-derived suppressor cell, a promising strategy to overcome resistance to immune checkpoint inhibitors. Front Immunol. (2020) 11:783. doi: 10.3389/fimmu.2020.00783, PMID: 32508809 PMC7249937

[26] Kim HR Park SM Seo SU Jung I Yoon HI Gabrilovich DI. The ratio of peripheral regulatory T cells to lox-1(+) polymorphonuclear myeloid-derived suppressor cells predicts the early response to anti-pd-1 therapy in patients with non-small cell lung cancer. Am J Respir Crit Care Med. (2019) 199:243–6. doi: 10.1164/rccm.201808-1502LE, PMID: 30339766 PMC6835091

[27] Möller M Orth V Umansky V Hetjens S Braun V Reißfelder C. Myeloid-derived suppressor cells in peripheral blood as predictive biomarkers in patients with solid tumors undergoing immune checkpoint therapy: systematic review and meta-analysis. Front Immunol. (2024) 15:1403771. doi: 10.3389/fimmu.2024.1403771, PMID: 38855104 PMC11157008

[28] Gaißler A Bochem J Spreuer J Ottmann S Martens A Amaral T. Early decrease of blood myeloid-derived suppressor cells during checkpoint inhibition is a favorable biomarker in metastatic melanoma. J Immunother Cancer. (2023) 11:e006802. doi: 10.1136/jitc-2023-006802, PMID: 37286306 PMC10254874

[29] Tomela K Pietrzak B Galus Ł Mackiewicz J Schmidt M Mackiewicz AA. Myeloid-derived suppressor cells (Mdsc) in melanoma patients treated with anti-pd-1 immunotherapy. Cells. (2023) 12:789. doi: 10.3390/cells12050789, PMID: 36899926 PMC10000540

[30] Maenhout SK Van Lint S Emeagi PU Thielemans K Aerts JL. Enhanced suppressive capacity of tumor-infiltrating myeloid-derived suppressor cells compared with their peripheral counterparts. Int J Cancer. (2014) 134:1077–90. doi: 10.1002/ijc.28449, PMID: 23983191

[31] Solito S Marigo I Pinton L Damuzzo V Mandruzzato S Bronte V. Myeloid-derived suppressor cell heterogeneity in human cancers. Ann N Y Acad Sci. (2014) 1319:47–65. doi: 10.1111/nyas.12469, PMID: 24965257

[32] Wang Z Zhou Q Zeng H Zhang H Liu Z Huang Q. Tumor-infiltrating il-17a(+) cells determine favorable prognosis and adjuvant chemotherapeutic response in muscle-invasive bladder cancer. Oncoimmunology. (2020) 9:1747332. doi: 10.1080/2162402x.2020.1747332, PMID: 32313725 PMC7153847

[33] Mikami S Mizuno R Kosaka T Saya H Oya M Okada Y. Expression of tnf-A and cd44 is implicated in poor prognosis, cancer cell invasion, metastasis and resistance to the sunitinib treatment in clear cell renal cell carcinomas. Int J Cancer. (2015) 136:1504–14. doi: 10.1002/ijc.29137, PMID: 25123505

[34] Zhang J Yan Y Cui X Zhang J Yang Y Li H. Ccl2 expression correlates with snail expression and affects the prognosis of patients with gastric cancer. Pathol Res Pract. (2017) 213:217–21. doi: 10.1016/j.prp.2016.12.013, PMID: 28215642

[35] Wang T Wei Y Tian L Song H Ma Y Yao Q. C-C motif chemokine ligand 5 (Ccl5) levels in gastric cancer patient sera predict occult peritoneal metastasis and a poorer prognosis. Int J Surg. (2016) 32:136–42. doi: 10.1016/j.ijsu.2016.07.008, PMID: 27398691

[36] Condamine T Mastio J Gabrilovich DI. Transcriptional regulation of myeloid-derived suppressor cells. J Leukoc Biol. (2015) 98:913–22. doi: 10.1189/jlb.4RI0515-204R, PMID: 26337512 PMC4661041

[37] Gabrilovich DI. Myeloid-derived suppressor cells. Cancer Immunol Res. (2017) 5:3–8. doi: 10.1158/2326-6066.Cir-16-0297, PMID: 28052991 PMC5426480

[38] Li Y Wu Z Ni C Li Y Wang P. Evaluation of the clinical significance of lymphocyte subsets and myeloid suppressor cells in patients with renal carcinoma. Discov Oncol. (2024) 15:512. doi: 10.1007/s12672-024-01405-2, PMID: 39347882 PMC11442913

[39] Lee CR Lee W Cho SK Park SG. Characterization of multiple cytokine combinations and tgf-B on differentiation and functions of myeloid-derived suppressor cells. Int J Mol Sci. (2018) 19:869. doi: 10.3390/ijms19030869, PMID: 29543758 PMC5877730

