# Supplementary material for: Pre-treatment monocytic myeloid-derived suppressor cells as predictive biomarkers for immune checkpoint inhibitor response in clear cell renal cell carcinoma
Source: Front Immunol. 2025 Aug 21;16:1641383. doi: 10.3389/fimmu.2025.1641383 (PMC12408328; doi:10.3389/fimmu.2025.1641383)
Supplement: Supplementary file 2 [file DataSheet2.docx]

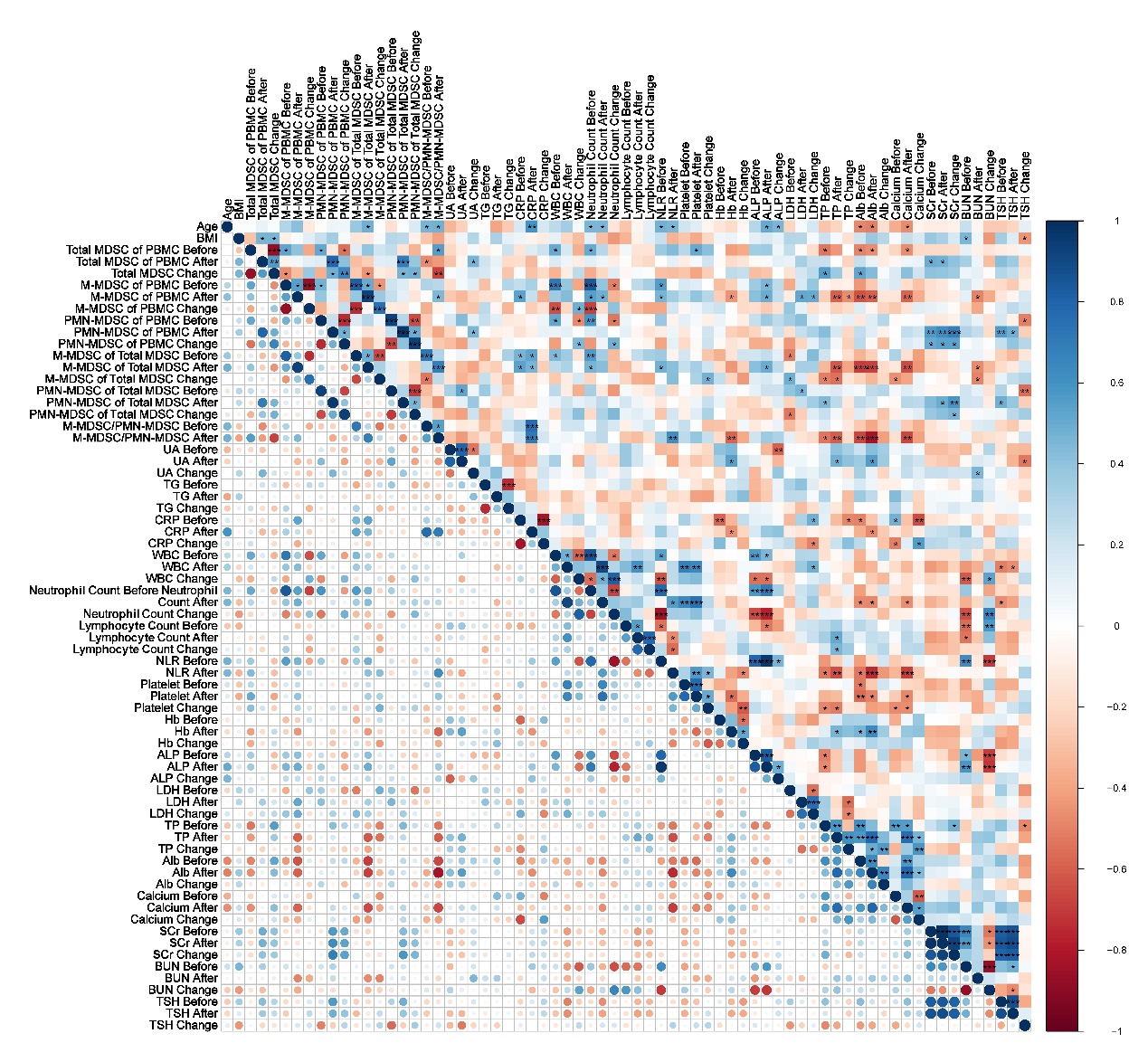


**Figure S1. Pearson correlation matrix of all numeric variables.**

BMI, body mass index; MDSC, myeloid-derived suppressor cell; M-MDSC, monocytic myeloid-derived suppressor cell; PMN-MDSC, polymorphonuclear myeloid-derived suppressor cell; UA, blood uric acid; TG, triglyceride; CRP, C-reactive protein; ALP, alkaline phosphatase; WBC, white blood cell; NLR, neutrophil-to-lymphocyte ratio; Hb, hemoglobin; LDH, lactate dehydrogenase; TP, total protein; Alb, albumin; SCr, serum creatinine; BUN, blood urea nitrogen; TSH, thyroid stimulating hormone. “*” = p < 0.1, “**” = p < 0.05, “***” = p < 0.01.
