# Supplementary material for: Pre-treatment monocytic myeloid-derived suppressor cells as predictive biomarkers for immune checkpoint inhibitor response in clear cell renal cell carcinoma
Source: Front Immunol. 2025 Aug 21;16:1641383. doi: 10.3389/fimmu.2025.1641383 (PMC12408328; doi:10.3389/fimmu.2025.1641383)
Supplement: Supplementary file 3 [file Table1.docx]

**Table S1. Mantel correlations between baseline MDSC indicators and clinical parameters.**

| **MDSCs Levels** | **Other Clinical Indicators** | **r** | **p.value** |
| --- | --- | --- | --- |
| Total MDSC Before | Age | -0.012 | 0.462 |
| Total MDSC Before | BMI | 0.196 | 0.075 |
| Total MDSC Before | UA Before | -0.014 | 0.45 |
| Total MDSC Before | TG Before | -0.002 | 0.414 |
| Total MDSC Before | CRP Before | -0.053 | 0.52 |
| Total MDSC Before | WBC Before | 0.260 | 0.049* |
| Total MDSC Before | Neutro Counts before | 0.314 | 0.064 |
| Total MDSC Before | Lympo Counts Before | 0.052 | 0.284 |
| Total MDSC Before | NLR Before | 0.195 | 0.103 |
| Total MDSC Before | Platelet Before | -0.020 | 0.466 |
| Total MDSC Before | Hb Before | -0.124 | 0.912 |
| Total MDSC Before | ALP Before | 0.130 | 0.146 |
| Total MDSC Before | LD Before | 0.070 | 0.224 |
| Total MDSC Before | TP Before | 0.407 | 0.008* |
| Total MDSC Before | Alb Before | 0.254 | 0.018* |
| Total MDSC Before | Calcium Before | 0.021 | 0.254 |
| Total MDSC Before | Cre Before | -0.063 | 0.54 |
| Total MDSC Before | BUN Before | 0.027 | 0.317 |
| Total MDSC Before | TSH Before | -0.105 | 0.804 |
| M-MDSC Before | Age | 0.134 | 0.181 |
| M-MDSC Before | BMI | -0.146 | 0.971 |
| M-MDSC Before | UA Before | -0.140 | 0.825 |
| M-MDSC Before | TG Before | -0.069 | 0.552 |
| M-MDSC Before | CRP Before | 0.112 | 0.21 |
| M-MDSC Before | WBC Before | 0.586 | 0.003* |
| M-MDSC Before | Neutro Counts before | 0.764 | 0.004* |
| M-MDSC Before | Lympo Counts Before | 0.311 | 0.037* |
| M-MDSC Before | NLR Before | 0.434 | 0.052 |
| M-MDSC Before | Platelet Before | -0.133 | 0.916 |
| M-MDSC Before | Hb Before | -0.059 | 0.572 |
| M-MDSC Before | ALP Before | 0.265 | 0.112 |
| M-MDSC Before | LD Before | 0.062 | 0.233 |
| M-MDSC Before | TP Before | 0.058 | 0.282 |
| M-MDSC Before | Alb Before | 0.053 | 0.279 |
| M-MDSC Before | Calcium Before | 0.197 | 0.128 |
| M-MDSC Before | Cre Before | -0.094 | 0.602 |
| M-MDSC Before | BUN Before | 0.131 | 0.23 |
| M-MDSC Before | TSH Before | -0.126 | 0.794 |
| PMN-MDSC Before | Age | 0.017 | 0.396 |
| PMN-MDSC Before | BMI | -0.125 | 0.889 |
| PMN-MDSC Before | UA Before | 0.025 | 0.325 |
| PMN-MDSC Before | TG Before | 0.104 | 0.211 |
| PMN-MDSC Before | CRP Before | -0.116 | 0.637 |
| PMN-MDSC Before | WBC Before | 0.347 | 0.031* |
| PMN-MDSC Before | Neutro Counts before | 0.478 | 0.021* |
| PMN-MDSC Before | Lympo Counts Before | 0.081 | 0.235 |
| PMN-MDSC Before | NLR Before | 0.251 | 0.158 |
| PMN-MDSC Before | Platelet Before | -0.103 | 0.823 |
| PMN-MDSC Before | Hb Before | -0.128 | 0.837 |
| PMN-MDSC Before | ALP Before | 0.111 | 0.208 |
| PMN-MDSC Before | LD Before | 0.410 | 0.009* |
| PMN-MDSC Before | TP Before | 0.020 | 0.394 |
| PMN-MDSC Before | Alb Before | 0.029 | 0.337 |
| PMN-MDSC Before | Calcium Before | -0.067 | 0.501 |
| PMN-MDSC Before | Cre Before | -0.122 | 0.93 |
| PMN-MDSC Before | BUN Before | 0.015 | 0.423 |
| PMN-MDSC Before | TSH Before | 0.084 | 0.223 |
| M-MDSC/Total MDSC Before | Age | 0.198 | 0.056 |
| M-MDSC/Total MDSC Before | BMI | -0.113 | 0.908 |
| M-MDSC/Total MDSC Before | UA Before | -0.095 | 0.786 |
| M-MDSC/Total MDSC Before | TG Before | -0.041 | 0.51 |
| M-MDSC/Total MDSC Before | CRP Before | 0.252 | 0.084 |
| M-MDSC/Total MDSC Before | WBC Before | 0.197 | 0.077 |
| M-MDSC/Total MDSC Before | Neutro Counts before | 0.321 | 0.036* |
| M-MDSC/Total MDSC Before | Lympo Counts Before | 0.037 | 0.344 |
| M-MDSC/Total MDSC Before | NLR Before | 0.142 | 0.153 |
| M-MDSC/Total MDSC Before | Platelet Before | -0.107 | 0.902 |
| M-MDSC/Total MDSC Before | Hb Before | 0.118 | 0.139 |
| M-MDSC/Total MDSC Before | ALP Before | 0.059 | 0.227 |
| M-MDSC/Total MDSC Before | LD Before | 0.091 | 0.166 |
| M-MDSC/Total MDSC Before | TP Before | 0.002 | 0.421 |
| M-MDSC/Total MDSC Before | Alb Before | -0.022 | 0.542 |
| M-MDSC/Total MDSC Before | Calcium Before | 0.148 | 0.165 |
| M-MDSC/Total MDSC Before | Cre Before | -0.116 | 0.865 |
| M-MDSC/Total MDSC Before | BUN Before | -0.042 | 0.492 |
| M-MDSC/Total MDSC Before | TSH Before | -0.122 | 0.853 |
| PMN-MDSC/Total MDSC Before | Age | 0.002 | 0.382 |
| PMN-MDSC/Total MDSC Before | BMI | -0.053 | 0.556 |
| PMN-MDSC/Total MDSC Before | UA Before | 0.095 | 0.206 |
| PMN-MDSC/Total MDSC Before | TG Before | 0.138 | 0.148 |
| PMN-MDSC/Total MDSC Before | CRP Before | -0.158 | 0.909 |
| PMN-MDSC/Total MDSC Before | WBC Before | 0.084 | 0.226 |
| PMN-MDSC/Total MDSC Before | Neutro Counts before | 0.180 | 0.124 |
| PMN-MDSC/Total MDSC Before | Lympo Counts Before | -0.071 | 0.592 |
| PMN-MDSC/Total MDSC Before | NLR Before | 0.070 | 0.165 |
| PMN-MDSC/Total MDSC Before | Platelet Before | -0.112 | 0.83 |
| PMN-MDSC/Total MDSC Before | Hb Before | -0.144 | 0.887 |
| PMN-MDSC/Total MDSC Before | ALP Before | -0.042 | 0.437 |
| PMN-MDSC/Total MDSC Before | LD Before | 0.496 | 0.005* |
| PMN-MDSC/Total MDSC Before | TP Before | 0.025 | 0.346 |
| PMN-MDSC/Total MDSC Before | Alb Before | 0.080 | 0.232 |
| PMN-MDSC/Total MDSC Before | Calcium Before | -0.092 | 0.673 |
| PMN-MDSC/Total MDSC Before | Cre Before | -0.139 | 0.994 |
| PMN-MDSC/Total MDSC Before | BUN Before | -0.062 | 0.5 |
| PMN-MDSC/Total MDSC Before | TSH Before | 0.202 | 0.13 |
| M-MDSC/PMN-MDSC Before | Age | 0.302 | 0.062 |
| M-MDSC/PMN-MDSC Before | BMI | -0.007 | 0.335 |
| M-MDSC/PMN-MDSC Before | UA Before | -0.097 | 0.664 |
| M-MDSC/PMN-MDSC Before | TG Before | -0.062 | 0.453 |
| M-MDSC/PMN-MDSC Before | CRP Before | 0.145 | 0.123 |
| M-MDSC/PMN-MDSC Before | WBC Before | -0.150 | 0.996 |
| M-MDSC/PMN-MDSC Before | Neutro Counts before | -0.119 | 0.911 |
| M-MDSC/PMN-MDSC Before | Lympo Counts Before | -0.132 | 0.88 |
| M-MDSC/PMN-MDSC Before | NLR Before | -0.092 | 0.953 |
| M-MDSC/PMN-MDSC Before | Platelet Before | -0.062 | 0.627 |
| M-MDSC/PMN-MDSC Before | Hb Before | 0.021 | 0.313 |
| M-MDSC/PMN-MDSC Before | ALP Before | -0.088 | 0.72 |
| M-MDSC/PMN-MDSC Before | LD Before | -0.053 | 0.541 |
| M-MDSC/PMN-MDSC Before | TP Before | 0.003 | 0.331 |
| M-MDSC/PMN-MDSC Before | Alb Before | -0.073 | 0.559 |
| M-MDSC/PMN-MDSC Before | Calcium Before | -0.022 | 0.411 |
| M-MDSC/PMN-MDSC Before | Cre Before | -0.096 | 0.921 |
| M-MDSC/PMN-MDSC Before | BUN Before | -0.143 | 0.958 |
| M-MDSC/PMN-MDSC Before | TSH Before | -0.090 | 0.744 |

BMI, body mass index; MDSC, myeloid-derived suppressor cell; M-MDSC, monocytic myeloid-derived suppressor cell; PMN-MDSC, polymorphonuclear myeloid-derived suppressor cell; UA, blood uric acid; TG, triglyceride; CRP, C-reactive protein; ALP, alkaline phosphatase; WBC, white blood cell; NLR, neutrophil-to-lymphocyte ratio; Hb, hemoglobin; LDH, lactate dehydrogenase; TP, total protein; Alb, albumin; SCr, serum creatinine; BUN, blood urea nitrogen; TSH, thyroid stimulating hormone.

**Table S2. Mantel test results showing correlations between changes in MDSC-related indicators and corresponding clinical parameter changes during treatment.**

| **MDSC Change Levels** | **Other Clinical Indicators Change Levels** | **r** | **p.value** |
| --- | --- | --- | --- |
| Total MDSC Change | UA Change | -0.120 | 0.878 |
| Total MDSC Change | TG Change | -0.021 | 0.462 |
| Total MDSC Change | CRP Change | 0.066 | 0.264 |
| Total MDSC Change | WBC Count Change | 0.084 | 0.225 |
| Total MDSC Change | Neutro Counts Change | 0.210 | 0.082 |
| Total MDSC Change | Lympo Counts Change | 0.180 | 0.018* |
| Total MDSC Change | Platelet Change | 0.281 | 0.056 |
| Total MDSC Change | Hb Change | 0.227 | 0.088 |
| Total MDSC Change | ALP Change | -0.126 | 0.795 |
| Total MDSC Change | LD Change | 0.037 | 0.337 |
| Total MDSC Change | TP Change | 0.068 | 0.253 |
| Total MDSC Change | Alb Change | 0.302 | 0.038* |
| Total MDSC Change | Calcium Change | -0.007 | 0.443 |
| Total MDSC Change | Cre Change | 0.232 | 0.073 |
| Total MDSC Change | BUN Change | 0.146 | 0.131 |
| Total MDSC Change | TSH Change | -0.086 | 0.694 |
| M-MDSC Change | UA Change | -0.041 | 0.574 |
| M-MDSC Change | TG Change | -0.064 | 0.561 |
| M-MDSC Change | CRP Change | 0.477 | 0.019* |
| M-MDSC Change | WBC Count Change | 0.288 | 0.077 |
| M-MDSC Change | Neutro Counts Change | 0.203 | 0.194 |
| M-MDSC Change | Lympo Counts Change | -0.004 | 0.464 |
| M-MDSC Change | Platelet Change | 0.071 | 0.295 |
| M-MDSC Change | Hb Change | -0.063 | 0.579 |
| M-MDSC Change | ALP Change | -0.118 | 0.699 |
| M-MDSC Change | LD Change | 0.394 | 0.017* |
| M-MDSC Change | TP Change | 0.182 | 0.146 |
| M-MDSC Change | Alb Change | -0.060 | 0.582 |
| M-MDSC Change | Calcium Change | 0.252 | 0.086 |
| M-MDSC Change | Cre Change | -0.091 | 0.514 |
| M-MDSC Change | BUN Change | 0.126 | 0.248 |
| M-MDSC Change | TSH Change | -0.115 | 0.714 |
| PMN-MDSC Change | UA Change | -0.158 | 0.905 |
| PMN-MDSC Change | TG Change | -0.070 | 0.581 |
| PMN-MDSC Change | CRP Change | -0.079 | 0.552 |
| PMN-MDSC Change | WBC Count Change | 0.215 | 0.132 |
| PMN-MDSC Change | Neutro Counts Change | 0.256 | 0.127 |
| PMN-MDSC Change | Lympo Counts Change | 0.101 | 0.111 |
| PMN-MDSC Change | Platelet Change | -0.140 | 0.761 |
| PMN-MDSC Change | Hb Change | -0.118 | 0.713 |
| PMN-MDSC Change | ALP Change | -0.055 | 0.551 |
| PMN-MDSC Change | LD Change | -0.240 | 0.958 |
| PMN-MDSC Change | TP Change | -0.100 | 0.7 |
| PMN-MDSC Change | Alb Change | -0.107 | 0.71 |
| PMN-MDSC Change | Calcium Change | -0.201 | 0.908 |
| PMN-MDSC Change | Cre Change | 0.427 | 0.035* |
| PMN-MDSC Change | BUN Change | 0.333 | 0.049* |
| PMN-MDSC Change | TSH Change | 0.075 | 0.297 |
| M-MDSC/Total MDSC Change | UA Change | -0.010 | 0.5 |
| M-MDSC/Total MDSC Change | TG Change | -0.133 | 0.775 |
| M-MDSC/Total MDSC Change | CRP Change | 0.416 | 0.011* |
| M-MDSC/Total MDSC Change | WBC Count Change | 0.071 | 0.308 |
| M-MDSC/Total MDSC Change | Neutro Counts Change | 0.001 | 0.423 |
| M-MDSC/Total MDSC Change | Lympo Counts Change | 0.052 | 0.236 |
| M-MDSC/Total MDSC Change | Platelet Change | 0.496 | 0.001* |
| M-MDSC/Total MDSC Change | Hb Change | 0.204 | 0.095 |
| M-MDSC/Total MDSC Change | ALP Change | -0.229 | 0.97 |
| M-MDSC/Total MDSC Change | LD Change | 0.196 | 0.103 |
| M-MDSC/Total MDSC Change | TP Change | 0.173 | 0.138 |
| M-MDSC/Total MDSC Change | Alb Change | 0.240 | 0.052 |
| M-MDSC/Total MDSC Change | Calcium Change | 0.330 | 0.021* |
| M-MDSC/Total MDSC Change | Cre Change | -0.146 | 0.815 |
| M-MDSC/Total MDSC Change | BUN Change | -0.116 | 0.701 |
| M-MDSC/Total MDSC Change | TSH Change | 0.004 | 0.442 |
| PMN-MDSC/Total MDSC Change | UA Change | -0.130 | 0.852 |
| PMN-MDSC/Total MDSC Change | TG Change | -0.106 | 0.681 |
| PMN-MDSC/Total MDSC Change | CRP Change | -0.143 | 0.754 |
| PMN-MDSC/Total MDSC Change | WBC Count Change | 0.114 | 0.237 |
| PMN-MDSC/Total MDSC Change | Neutro Counts Change | 0.170 | 0.174 |
| PMN-MDSC/Total MDSC Change | Lympo Counts Change | 0.082 | 0.158 |
| PMN-MDSC/Total MDSC Change | Platelet Change | -0.103 | 0.719 |
| PMN-MDSC/Total MDSC Change | Hb Change | -0.142 | 0.798 |
| PMN-MDSC/Total MDSC Change | ALP Change | -0.092 | 0.676 |
| PMN-MDSC/Total MDSC Change | LD Change | -0.229 | 0.961 |
| PMN-MDSC/Total MDSC Change | TP Change | -0.193 | 0.931 |
| PMN-MDSC/Total MDSC Change | Alb Change | -0.109 | 0.742 |
| PMN-MDSC/Total MDSC Change | Calcium Change | -0.211 | 0.937 |
| PMN-MDSC/Total MDSC Change | Cre Change | 0.230 | 0.131 |
| PMN-MDSC/Total MDSC Change | BUN Change | 0.161 | 0.183 |
| PMN-MDSC/Total MDSC Change | TSH Change | 0.258 | 0.082 |

MDSC, myeloid-derived suppressor cell; M-MDSC, monocytic myeloid-derived suppressor cell; PMN-MDSC, polymorphonuclear myeloid-derived suppressor cell; UA, blood uric acid; TG, triglyceride; CRP, C-reactive protein; ALP, alkaline phosphatase; WBC, white blood cell; NLR, neutrophil-to-lymphocyte ratio; Hb, hemoglobin; LDH, lactate dehydrogenase; TP, total protein; Alb, albumin; SCr, serum creatinine; BUN, blood urea nitrogen; TSH, thyroid stimulating hormone.
